# Clamp Plate

**Published:** 1887-07

**Authors:** W. G. Browne


ARTICLE VI.

### CLAMP PLATE.

BY DR. W. G. BROWNE.

The mean between extremes is most to be desired and is most productive of good results. Acknowledging the great merit of bridge-work proper and the demerits of the plate covering the roof of the mouth, I have made a compromise between the two which embraces the ability to remove and clean as with the plate, and the convenience of the bridge in not becoming an obstruction in the mouth—a plan better adapted to the skill of the average practitioner and the purse of the average patient. I do not claim that my plan can be used in all cases, but principally for bicuspids and molars, and sometimes for incisors.

For a description I refer you to a typical case. Here is a model and piece that has been worn seven years. You see the two right superior bicuspids are gone, with the first and second molars remaining; that the artificial teeth are ground to fit snugly between the molar and cuspid, slightly overlapping the molar, and a small plate of rubber just large enough to saddle the ridge and extend back along the palatine sides of the molars and clamp the second molar. This plate is about one-fourth of an inch wide, and is not an inconvenience to the patient. Your attention is specially directed to one of the essential things requisite to success, viz., the stability of the piece is due largely to the clamp on the second molar. You will observe that I do not care for the piece to fit closely along the palatine side of the molars, but that it does bear on the palatine and buccal surfaces of the second molar, thus clamping it. Never fit a piece in such manner that the change of position of the natural teeth can affect the stability of the artificial teeth.

It often happens that the wisdom tooth is in position and cone shaped in such a way that it is not available for clamping to; in such cases I use the first molar alone, grinding the artificial bicuspid to overlap it on the buccal side, thus making one bearing for the clamp, and extending the piece to a little beyond the opposite point to get the other. A number of pieces of this kind have been in use for a long time, and are eminently successful, the patients wearing them expressing the greatest satisfaction.

We find many cases where several teeth are missing from the lower jaw—for example, one bicuspid and two molars, with third molar in position. This clamping process is available here by putting it on the molar and partially around the bicuspid, but not necessary to clamp it.

I present also for your inspection a piece with only one tooth on it, to show you how small and light the appliance can be made, and which will also show how incisors and cuspids can be inserted if the other teeth are in favorable condition.

I have no doubt that some will think that there is not much new in this. I have only to say that it is new to me and I have never seen a case made by any one else, and if this system has been known to any of you, why have you not published it?

As before intimated, the bridge-work is good, and so are the prices obtained for it; but how many of the average dentists can make a durable piece of it, and how many of our patients can pay for it? But here is a system that can be worked by any good dentist, and paid for by any one, and can be removed at will and cleaned.—*Proceedings of Georgia State Dental Society—Southern Dental Journal.*

